# Supplementary material for: Heterologous Expression of a Ferritin Homologue Gene PpFer1 from Prunus persica Enhances Plant Tolerance to Iron Toxicity and H2O2 Stress in Arabidopsis thaliana
Source: Plants (Basel). 2023 Dec 7;12(24):4093. doi: 10.3390/plants12244093 (PMC10747543; doi:10.3390/plants12244093)
Supplement: Supplementary file 1 [file plants-12-04093-s001.zip › plants-2715087-supplementary/Supplementary Figures.pdf]

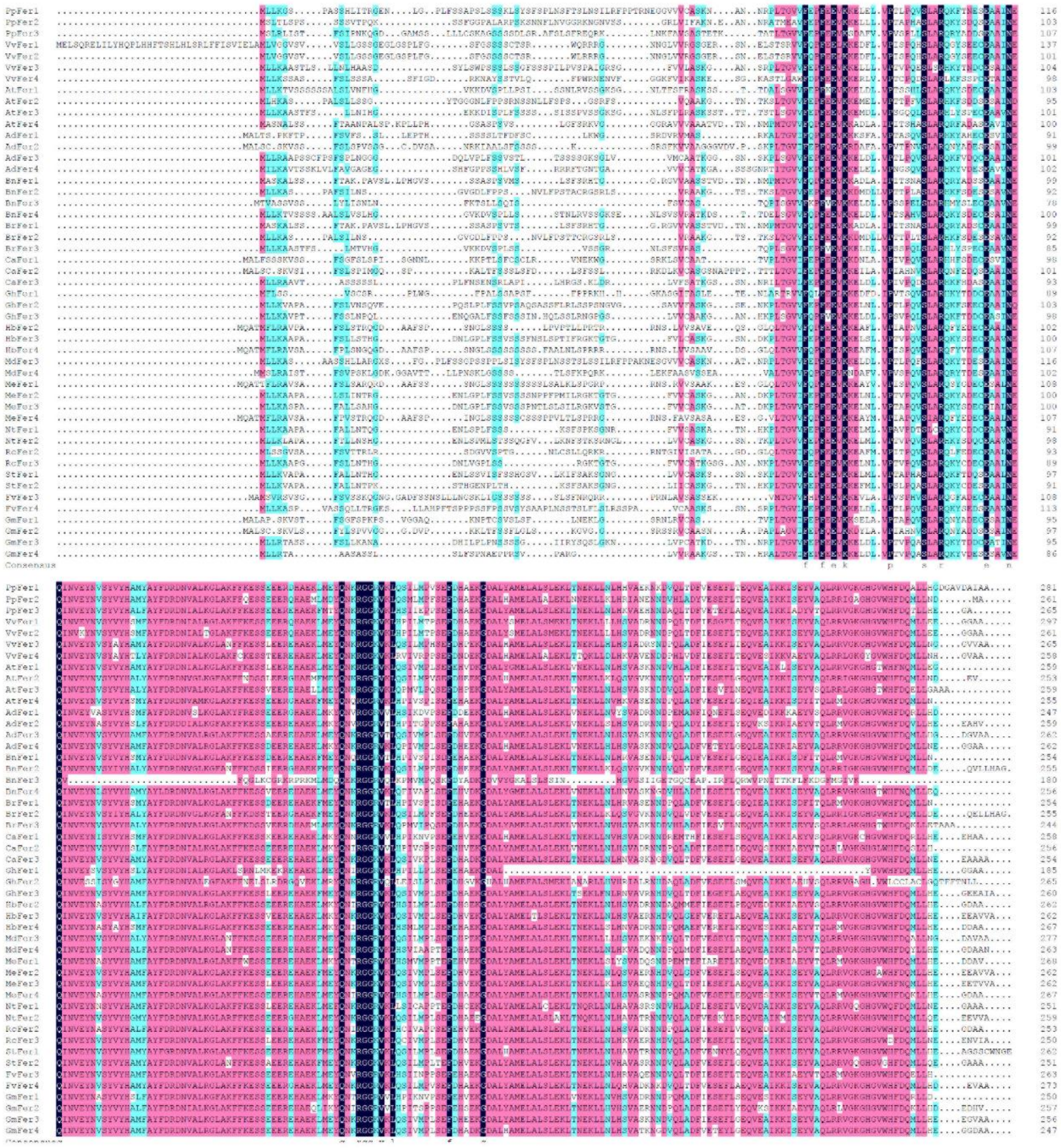

Figure S1: Amino acid alignment of known plant Ferritin homologues;

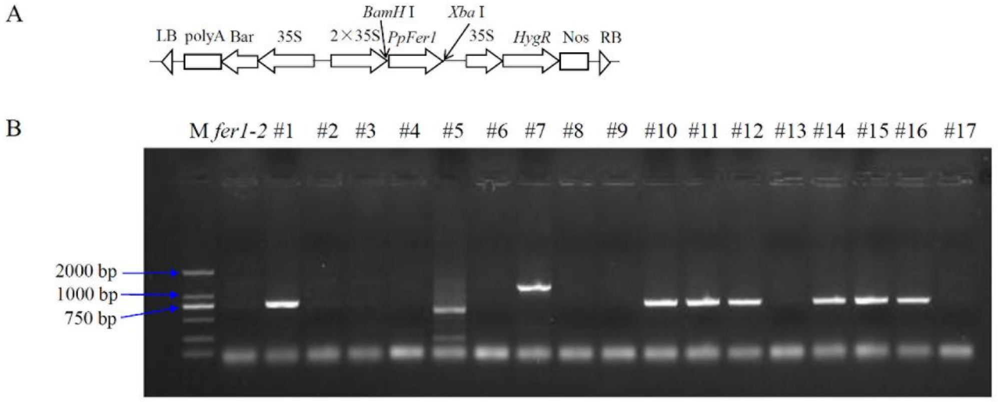

Figure S2: Generation of *PpFer1* over-expression transgenic *Arabidopsis* seedlings. (A) Construction of recombinant plasmid pBH-*PpFer1*. (B) PCR verification of *PpFer1* in T1 generation *fer1-2*/35S::*PpFer1* lines. Note: M, standard DL2000 DNA ladder (Takara, Dalian, China); Table S1: Specific primers used for quantitative RT-PCR
