# Supplementary material for: Heterologous Expression of a Ferritin Homologue Gene PpFer1 from Prunus persica Enhances Plant Tolerance to Iron Toxicity and H2O2 Stress in Arabidopsis thaliana
Source: Plants (Basel). 2023 Dec 7;12(24):4093. doi: 10.3390/plants12244093 (PMC10747543; doi:10.3390/plants12244093)
Supplement: Supplementary file 1 [file plants-12-04093-s001.zip › plants-2715087-supplementary/Supplementary Table S1.pdf]

Table S1. Specific primers used for quantitative RT-PCR.

Gene	Primer sequence (5’ to 3’)	Product size（bp）
PpFer1	[F]: ACGCGGTGGAAGAGTGAAAT	219
	[R]: GCTTCCACCTGCTCAGTCAA	
PpFer2	[F]: ATGCTGAAAAGGGTGAGGCA	231
	[R]: AGTGCCAAACACCATGTCCT	
PpFer3	[F]: CTTGCTGTGCTCAAAGGCTG	202
	[R]: TAGCAGAGGGGACACAGGAA	
